# Metabolomics for Biomarker Discovery: Key Signatory Metabolic Profiles for the Identification and Discrimination of Oat Cultivars

**DOI:** 10.3390/metabo11030165

**Published:** 2021-03-12

**Authors:** Chanel J. Pretorius, Fidele Tugizimana, Paul A. Steenkamp, Lizelle A. Piater, Ian A. Dubery

**Affiliations:** Research Centre for Plant Metabolomics, Department of Biochemistry, University of Johannesburg, P.O. Box 524, Auckland Park, Johannesburg 2006, South Africa; 201423600@student.uj.ac.za (C.J.P.); ftugizimana@uj.ac.za (F.T.); psteenkamp@uj.ac.za (P.A.S.); lpiater@uj.ac.za (L.A.P.)

**Keywords:** *Avena sativa*, cultivar distinction, liquid chromatography, mass spectrometry, metabolomics, multivariate data analysis, oat, secondary metabolites

## Abstract

The first step in crop introduction—or breeding programmes—requires cultivar identification and characterisation. Rapid identification methods would therefore greatly improve registration, breeding, seed, trade and inspection processes. Metabolomics has proven to be indispensable in interrogating cellular biochemistry and phenotyping. Furthermore, metabolic fingerprints are chemical maps that can provide detailed insights into the molecular composition of a biological system under consideration. Here, metabolomics was applied to unravel differential metabolic profiles of various oat (*Avena sativa*) cultivars (Magnifico, Dunnart, Pallinup, Overberg and SWK001) and to identify signatory biomarkers for cultivar identification. The respective cultivars were grown under controlled conditions up to the 3-week maturity stage, and leaves and roots were harvested for each cultivar. Metabolites were extracted using 80% methanol, and extracts were analysed on an ultra-high performance liquid chromatography (UHPLC) system coupled to a quadrupole time-of-flight (qTOF) high-definition mass spectrometer analytical platform. The generated data were processed and analysed using multivariate statistical methods. Principal component analysis (PCA) models were computed for both leaf and root data, with PCA score plots indicating cultivar-related clustering of the samples and pointing to underlying differential metabolic profiles of these cultivars. Further multivariate analyses were performed to profile differential signatory markers, which included carboxylic acids, amino acids, fatty acids, phenolic compounds (hydroxycinnamic and hydroxybenzoic acids, and associated derivatives) and flavonoids, among the respective cultivars. Based on the key signatory metabolic markers, the cultivars were successfully distinguished from one another in profiles derived from both leaves and roots. The study demonstrates that metabolomics can be used as a rapid phenotyping tool for cultivar differentiation.

## 1. Introduction

Food demand has been rapidly increasing with the overall growth in the world population, which is expected to reach around 9.7 billion by the year 2050 [1]. Now more than ever, crop improvement and plant breeding studies have become imperative in ensuring food security and sustainability [2]. The primary step involved in plant breeding, inspection, registration, trade and seed production requires the identification of cultivars and varieties, and therefore a rapid and effective method for cultivar fingerprinting is required [3]. Over the years, plant breeding has been greatly improved for unravelling the molecular basis of complex traits using genomic analyses and next-generation sequencing methods [4]. Currently, plant breeding methods have integrated phenotypic traits with a range of marker-assisted selection techniques to more efficiently determine trait outcomes [5]. Although genetic markers have been at the forefront of plant breeding efforts, many limitations have restricted the use thereof in analysing complexities arising from genotype × environment interactions (environmental plasticity), polygenic inheritance and epistasis (which is referred to as the action of one gene on another) [6].

**Figure 1:**
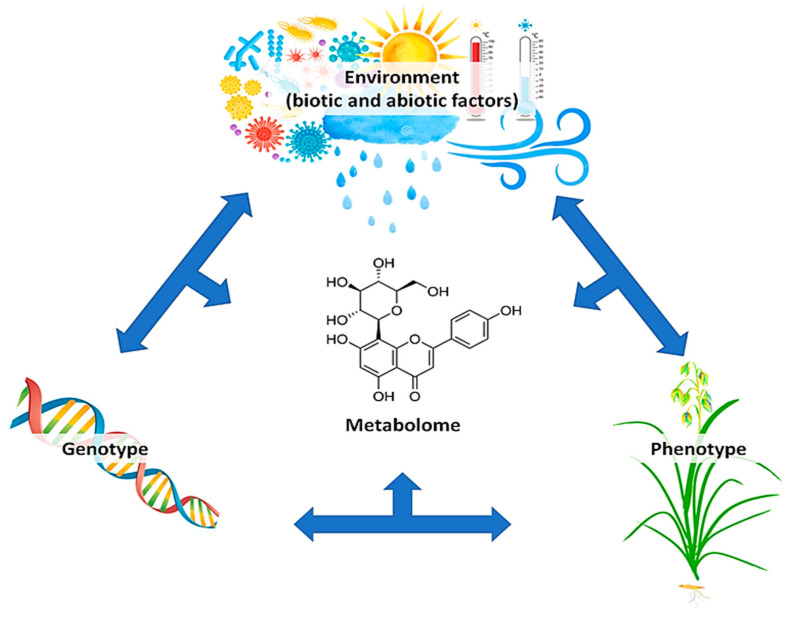
Triangular arrangement illustrating the genotype × environment × phenotype interactions, with the metabolome at the core, bridging the gap between the genotype and phenotype. The metabolome is the final recipient of biological information flow and carries imprints of genetic and environmental factors. It is more sensitive to perturbations in both metabolic fluxes and enzyme activity than either the transcriptome or proteome and is thus a reflection of the phenotype. Quantitative, global measurements of the metabolome therefore provide an exploration of cellular metabolism, revealing patterns and functional signatures of the biochemical landscape and cellular physiology of the system under consideration [8,9,10].

Metabolomics, a systems biology approach to interrogate cellular biochemistry and metabolism, offers unique possibilities that can be incorporated into unravelling these complexities to gain a genotype × metabolite × phenotype understanding that can be applied in plant breeding. The phenotype is an observable reflection that results from complex interactions between the genotype and the environment, with the former also able to prime multiple phenotypes [7]. These interactions can result in various success rates in reproduction and cause subsequent alterations in the genotype, as summarised in the abridged illustration (Figure 1). To bridge the gap between the genotype and phenotype, metabolomics was proposed as a means to provide insight into how genotypic variation affects phenotypic diversity in plants [8,9,10].

Metabolomics, defined as the comprehensive qualitative and quantitative analysis of all metabolites in a biological system, is an established omics technology that holds promise in agricultural research; therefore, metabolomics has become an indispensable tool in various plant sciences studies [11,12]. Due to the diverse and large variety of metabolites found in plants, an extensive array of analytical techniques has been developed to obtain sufficient coverage for plant metabolomics. Liquid chromatography–mass spectrometry (LC–MS)-based plant metabolomics, compared to that of gas chromatography–mass spectrometry (GC–MS) and nuclear magnetic resonance (NMR), has been advantageous in detecting a wide range of secondary metabolites with higher sensitivity and selectivity (compared to NMR) and has the ability to detect and identify a broader range of compounds whilst being less time-consuming in the preparation of samples (compared to GC–MS which requires derivatisation) [13,14,15].

In the past, metabolomics has proven crucial for studying plant x environment interactions (e.g., adaptive responses towards biotic and abiotic stresses) and has been applied in metabolomics-assisted breeding of crops. So far, great progress has been made in the development of metabolomics tools for crop improvement. However, there are still bottlenecks with regards to analytical technologies and tools used for data mining and interpretation. Some of these limit metabolome coverage, the maximisation of metabolomics data and the annotation of extracted metabolites [16,17,18]. In plants, metabolites are known to play important roles in crop yield, nutritional quality, growth and development, as well as in plant defence against environmental stresses [19,20]. Different metabolomic applications have therefore been developed to elucidate plant responses and mechanisms under different conditions to determine metabolic profiles for use in crop improvement [16]. As such, metabolomics allows the predictive discovery of biomarkers, independent of genetic and environmental variation. These metabolite biomarkers provide invaluable information on biochemical mechanisms that underly phenotypic traits and can be used in the development of targeted methods for breeding programmes [17,21,22]. Plant metabolites are increasingly incorporated into breeding programmes for the prediction of phenotypic traits and thus provide an early detection tool for identifying favourable traits.

In this study, metabolomics tools and approaches were applied in order to develop a profiling methodology able to discriminate between various oat (*Avena sativa* L.) cultivars. Oat belongs to the monocotyledonous *Poaceae* family along with other cereals such as wheat, rice, barley, rye, maize, sorghum and millet [23]. Of these, oat has recently attracted renewed interest due to numerous health and nutritional benefits involved in both human and livestock consumption [24,25]. Oat is also considered a superior cereal crop due to its hardiness and ability to thrive and withstand environmentally poor conditions where other cereals seem to be lacking [26].

## 2. Results

### 2.1. Differential Chromatographic–Mass Spectrometric Analyses of Respective Oat Cultivars

**Figure 2:**
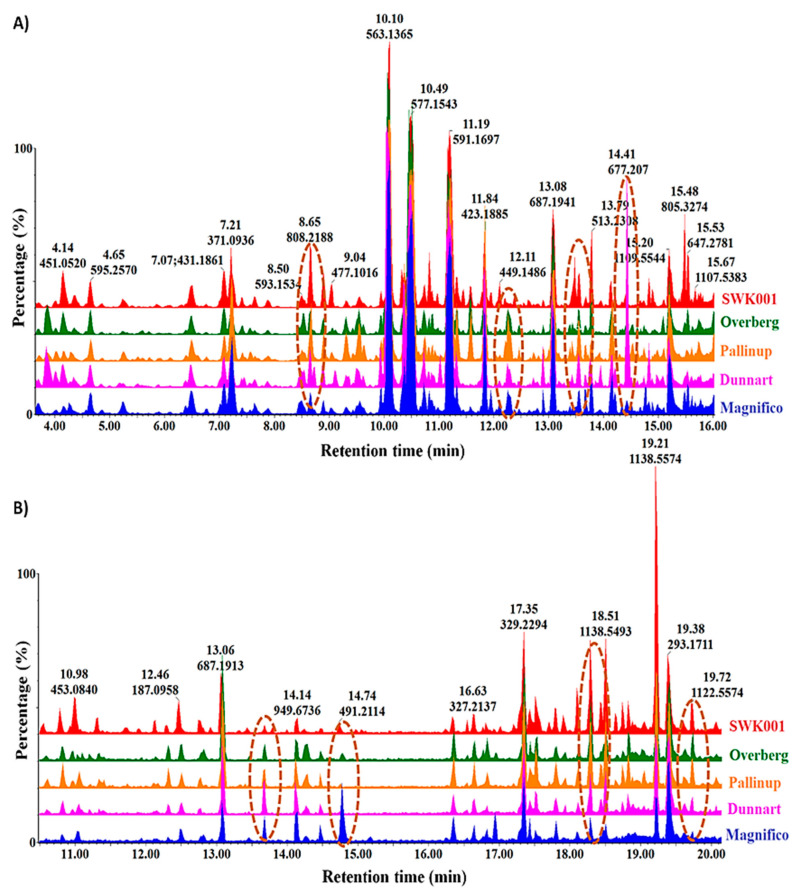
Ultra-high performance liquid chromatography (UHPLC) coupled to mass spectrometric (MS) detection. The figure compares base peak intensity (BPI) MS chromatograms of methanol (**A**) leaf and (**B**) root extracts from five oat cultivars (SWK001, Overberg, Pallinup, Dunnart and Magnifico) at the seedling stage. These represent the chromatographic separation based on the polarity of the different compounds in negative ionisation mode (ESI–), separated on an HSS T3 reverse-phase column. The dashed oval structures point out some cultivar-exclusive variations that illustrate differences in the phytochemical profiles of the cultivars.

Methanolic extracts of leaf and root tissues of the respective cultivars were separated on an ultra-high performance liquid chromatography system coupled to a quadrupole time-of-flight high-definition mass spectrometer (UHPLC–qTOF–MS) and detected in both positive and negative electrospray ionisation (ESI) modes. Initial optimisation studies indicated that the majority of extractable metabolites ionised more effectively in the ESI (−) mode; accordingly, only these datasets are further presented and illustrated. The chromatographically distinct base peak intensity (BPI) chromatograms of leaf and root extracts (Figure 2A,B) provide a visual presentation/description of the similarities and differences between the respective cultivars and reflect the complexity of their metabolic profiles. Although chromatography is extremely useful in separating the components based on their polarity, and high-definition mass spectrometry enables accurate mass determination in order to generate empirical formulae to aid in compound annotation, further chemometric analyses were performed to obtain biologically useful information.

### 2.2. Chemometric Analyses for Profiling the Oat Cultivar Metabolomes

**Figure 3:**
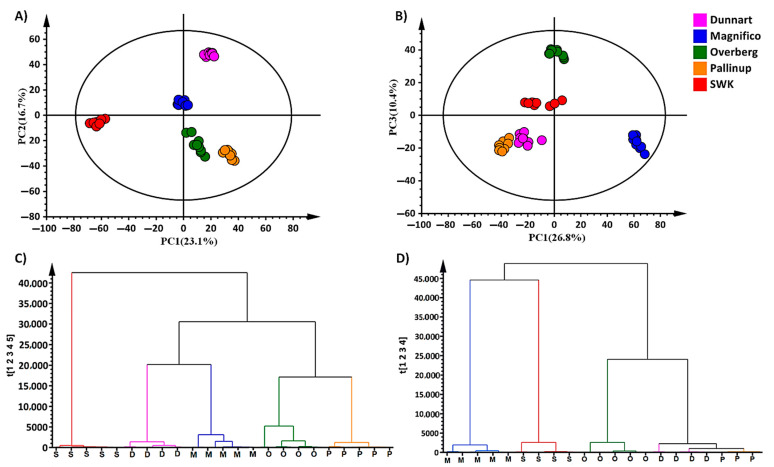
Principal component analysis (PCA) of five oat cultivars with the corresponding hierarchical cluster analysis (HiCA) dendrograms. PCA score plots indicate the clustering and general grouping among the five cultivars (Overberg—O, Pallinup—P, Dunnart—D, Magnifico—M, and SWK001—S) extracted from (**A**) leaf and (**B**) root tissues, analysed in ESI(–) mode. The HiCA dendrogram (**C**) shows the hierarchical structure of the leaf data indicating that ‘Pallinup’ is phytochemically more similar to ‘Overberg’, and ‘Dunnart’ to ‘Magnifico’. In comparison, ‘SWK001’ is the most different from the other cultivars concerning their leaf metabolic profiles. The HiCA dendrogram (**D**) illustrates the root metabolic profiles; ‘Pallinup’ and ‘Dunnart’ are similar and cluster closely with ‘Overberg’. In comparison, ‘Magnifico’ and ‘SWK001’ are metabolically different from the other three cultivars based on the extracted profiles.

Due to the complexity and multi-dimensionality of metabolomic data, appropriate statistical and chemometric tools are required to obtain chemical information and convert it into biological knowledge [27]. Chemometrics is the science of extracting useful information from complex datasets through pattern recognition and machine learning algorithms [28,29]. Principal component analysis (PCA) is a multivariate technique that increases the interpretability and minimises the loss of biological information by reducing the dimensionality of complex datasets [30]. The underlying structures and characteristics of the data are thus revealed by this unsupervised, explorative method. The illustrated PCA models (Figure 3) show distinct clustering of the five respective cultivars (Magnifico, Dunnart, Pallinup, Overberg and SWK001), which points to underlying differential metabolic profiles from the leaf (Figure 3A) and root (Figure 3B) tissues. The model illustrates both similarities and differences within (PC2/3) and between (PC1) the cultivar groupings. This differential clustering revealed by PCA relates to the differences previously visualised by the chromatographic separation (Figure 2).

In addition to PCA modelling, another unsupervised technique, namely, hierarchical cluster analysis (HiCA), was used to cluster high-dimensional data into a dendrogram based on the dissimilarity and similarity of the samples [31]. In a bottom-up representation (Figure 3C,D), the algorithm clusters each observation based on their differences and further proceeds by joining the most similar clusters at each step in an iterative manner. The resulting dendrogram illustrates that the metabolic profiles of the leaf tissues (Figure 3C) of ‘Magnifico’ and ‘Dunnart’ appear to be closely related; this is similar in the case of ‘Pallinup’ and ‘Overberg’, with ‘SWK001’ appearing to be most metabolically different and clustering at the far left. The cultivars also cluster separately based on their profiles extracted from root tissues (Figure 3D); in this case, however, ‘Dunnart’ and ‘Pallinup’ seem to be most similar metabolically and, in turn, are grouped with ‘Overberg’. ‘Magnifico’ and ‘SWK001’, in this case, are the most metabolically different from the other cultivars and similar to each other. It is of interest that the unsupervised, explorative method not only underscored differences between cultivars but also highlighted differences between the extracts from roots and leaves of these cultivars. The source-to-sink model describes differences between various plant tissues based on their environment as well as the synthesis and transport of various nutrients required for growth and development [32]. Source tissues are often described as net exporters of resources required for plant growth, such as carbon or nitrogen, while sink tissues are net importers responsible for resource absorption. Mature leaves are net sources of carbon but sink for nitrogen, while root tissues are net sources of nitrogen but sink for carbon [33]. Another example contributing to the differences among the respective tissues is the presence of secondary metabolites. These may be uniquely synthesised by either the leaves or roots, such as the case for avenacins, which are triterpenoid saponins found in roots whilst the leaves contain steroidal saponins known as avenacosides. Both compounds serve a similar purpose but are respectively confined to the various plant tissues [34]. Once differences are apparent, more in-depth information can be obtained through supervised methods such as orthogonal projection to latent structures discriminant analysis (OPLS-DA).

**Figure 4:**
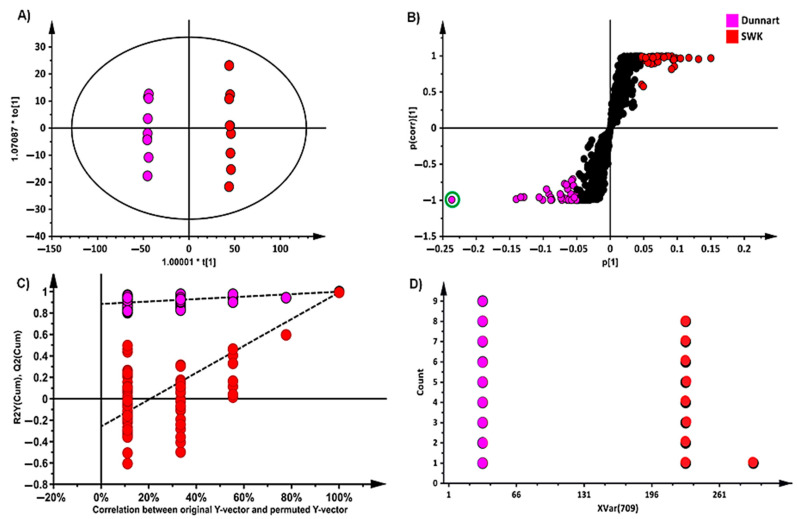
An orthogonal projection to latent structures discriminant analysis (OPLS-DA) model of two representative cultivars: ‘Dunnart’ and ‘SWK001’. (**A**) A score plot summarising the relationship among different datasets to visualise group clustering between the ‘Dunnart’ and ‘SWK001’ cultivars based on their leaf-extracted metabolic profiles obtained in ESI (−) MS mode (R^2^ = 0.998, Q^2^ = 0.971, CV-ANOVA *p*-value = 5.8941 × 10^−14^). (**B**) The corresponding loadings S-plot. The pink and red circles indicate the outlier values (*p*(corr) [1] ≥0.5, ≤−0.5) and covariance of *p* [1] ≥0.05, ≤−0.05) in the S-plot, indicating statistically significant ions that are possible discriminatory variables between the two cultivars. (**C**) Permutation test plot (n = 100) for the OPLS-DA model (**A**) was used to validate the predictive capability. (**D**) Dot plot illustrating strong discrimination between the cultivars for the selected variable (circled in green on the S-plot) as there is no overlap between the groups.

OPLS-DA modelling showed sample classification in the score space between different experimental groups, as depicted in Figure 4A. With the ‘SWK001’ and ‘Dunnart’ cultivars, clear clustering and group separation are shown. As a supervised method, OPLS-DA is often considered a model that is prone to overfitting data; therefore, rigorous model validation methods are used to ensure the validity and reliability of the computed model [35]. The reliability of the models was tested using cross-validation analysis of variance (CV-ANOVA) where the significant models had *p*-values of <0.05. Furthermore, the performance of the OPLS-DA models was evaluated using receiver operator characteristic (ROC) models where perfect classification was depicted as the ROC curve passed through the top left corner, indicating perfect sensitivity and specificity (Supplementary Figure S1). Finally, permutation tests were performed where the OPLS-DA models were statistically shown to be better than the generated permutation models with the R^2^ and Q^2^ being higher for the OPLS-DA model (Figure 4C). The loadings S-plot (Figure 4B) was used to target and select statistically significant discriminatory ions among the different cultivars. Furthermore, each selected variable from the S-plot was evaluated using a dot plot (Figure 4D) that computes each observation as a unit and subsequently sorts each component into “bins” that represent sub-ranges. Strong discriminating variables show no overlap between the groups, as can be seen in Figure 4D. OPLS-DA models and their corresponding loadings S-plots were similarly constructed for all cultivars for both leaf and root tissue extracts (20 in total for each tissue type—model infographics are available on request).

### 2.3. Differential Metabolic Profiles Based on Discriminatory Ions

**Figure 5:**
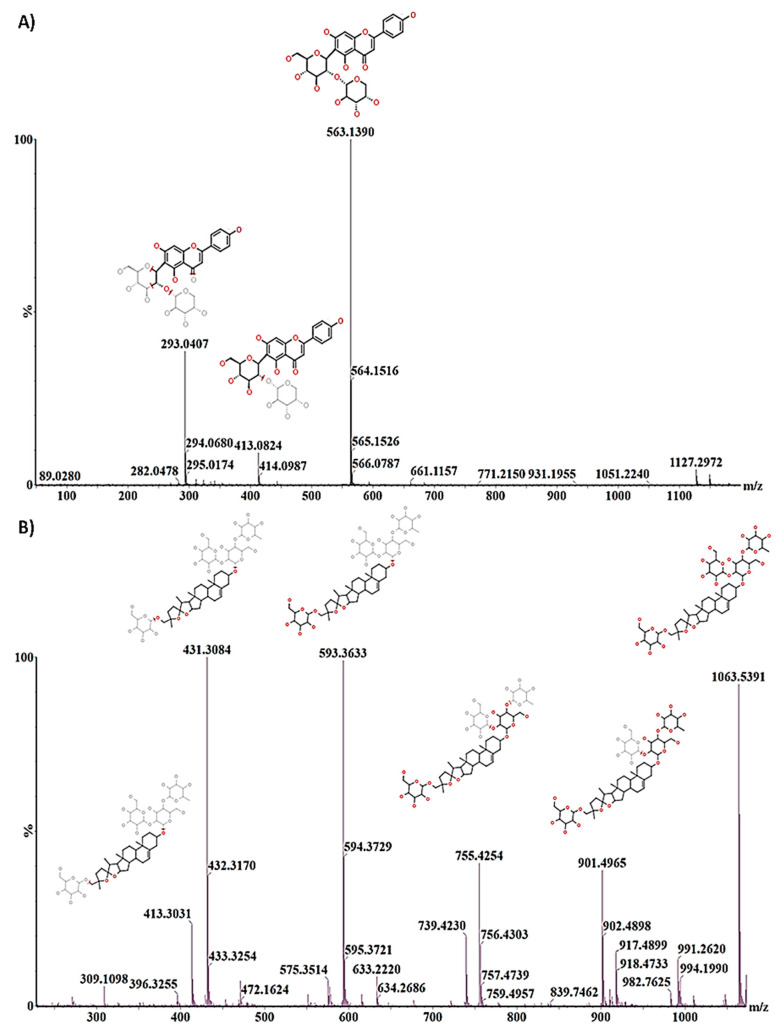
Representation of the use of fragmentation patterns for the annotation of secondary metabolites. (**A**) Isovitexin 2″-*O*-arabinoside showing the parent ion at *m*/*z* 563 and two diagnostic fragment ions at *m*/*z* 413 and 293. (**B**) Avenacoside A at an *m/z* of 1063 showing diagnostic fragments (*m*/*z* 901, 755, 593, 431 and 413) with their structural changes that aid in the structural identification of the metabolite. The fragmentation spectra enable confirmation of the elemental composition and provide useful hints to elucidate possible structural information by evaluating fragmentation patterns yielded at different collision energies, MS^E^.

Following the selection of discriminant ions from the respective loadings S-plots, a list of putatively identified metabolites was compiled and is presented in Table 1. The statistically significant variables were annotated as described in experimental Section 4.6. The possible chemical structures were then explored by further inspection of the generated fragmentation patterns under various collision energies (MS^E^) (Figure 5). The annotated metabolites thus represent the discriminatory compounds that allowed for differentiation among the different cultivars. Datasets from all five cultivars were compared to one another. In Table 1, the asterisks within the coloured cells indicate metabolites that were discriminatory for the respective cultivars compared to the other four cultivars and are indicated when detected against one or more of the other cultivars. These compounds were placed in the following metabolite classes: carboxylic acids, amino acids, fatty acids, phenolics and flavonoids. In addition, a steroidal saponin (avenacoside A) was annotated in extracts from leaves and a triterpenoid saponin (avenacin A-1) in extracts of roots.

**Figure 6:**
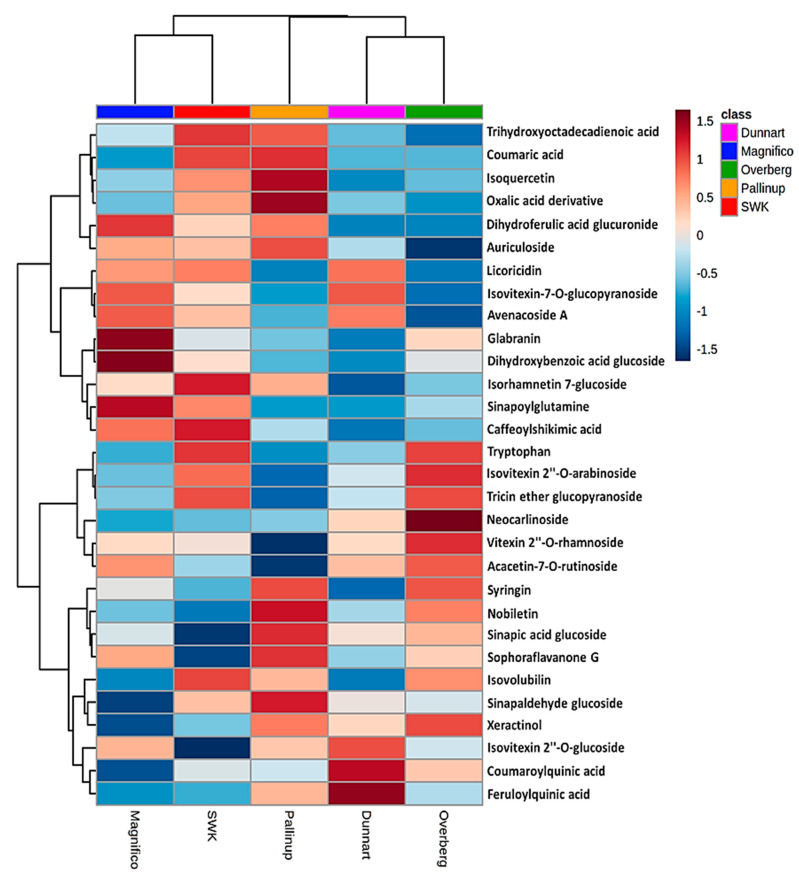
Individual peak intensities of annotated metabolites from oat leaves using heatmap analysis (Pearson distance and Ward’s linkage rule) of the five cultivars ‘Overberg’, ‘Pallinup’, ‘Dunnart’, ‘Magnifico’ and ‘SWK001’. The mean peak intensities of each annotated metabolite are shown after Pareto scaling of the data. Values higher than the averages are shown in red and lower values in blue, with each row representing discriminant features and each column representing the respective cultivars.

**Figure 7:**
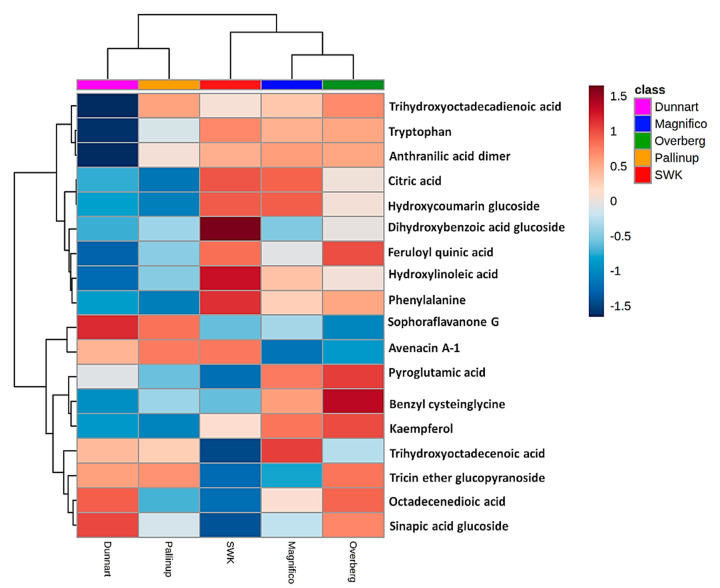
Individual peak intensities of annotated metabolites from oat roots using heatmap analysis (Pearson distance and Ward’s linkage rule) of the five cultivars ‘Overberg’, ‘Pallinup’, ‘Dunnart’, ‘Magnifico’ and ‘SWK001’. The mean peak intensities of each annotated metabolite are shown after Pareto scaling of the data. Values higher than the averages are indicated in red and lower values in blue, with each row representing discriminant features and each column representing the respective cultivars.

**Figure 8:**
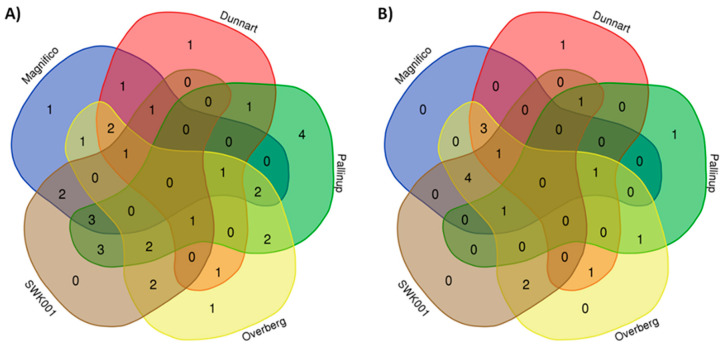
Venn diagrams displaying the partial overlap and differences of statistically significant variables selected from the OPLS-DA models for (**A**) leaves and (**B**) roots. The numerical values in the diagram represent the discriminatory metabolites (Table 1) that are unique to the respective cultivars and, conversely, also shared between the cultivars.

Data visualisation tools were used to illustrate the magnitude and presence of the respective metabolites in various cultivars with heatmap analysis (Figure 6 and Figure 7). Here, the average integrated peak areas of the respective metabolites were used to construct heatmaps using statistical analysis software available on MetaboAnalyst https://www.metaboanalyst.ca/ (accessed on 12 March 2021) [36]. Five well-defined clusters are illustrated that relate to the five different experimental groups. These infographics show clear differences among the cultivars with respect to their various metabolic profiles. These profiles could prove useful in not only discriminating among the various cultivars but also providing useful information on possible links to stress resistance or susceptibility capabilities between them. Among the identified metabolites (Table 1), the differential metabolic profiles based on discriminatory ions present in the hydromethanolic extracts of the various cultivars were as follows: ‘Magnifico’ contained 9 flavonoids, 5 phenols and avenacoside A in the leaves, and 3 amino acids, 1 carboxylic acid, 3 fatty acids, 2 flavonoids and 1 phenol in the roots. ‘Dunnart’, on the other hand, had 7 flavonoids, 4 phenols and avenacoside A in the leaves, and 1 amino acid derivative, 1 carboxylic acid, 2 fatty acids, 2 flavonoids, 1 phenol and avenacin A-1 in the roots. ‘Pallinup’ showed a metabolic profile containing 3 fatty acids, 9 flavonoids and 6 phenols in the leaves, and 1 amino acid, 1 fatty acid, 1 flavonoid, 1 phenol and avenacin A-1 in the roots. ‘Overberg’ had 1 amino acid, 10 flavonoids, 4 phenols and avenacoside A in the leaves, and 3 amino acids, 1 carboxylic acid, 3 fatty acids, 2 flavonoids and 5 phenols in the roots. Lastly, ‘SWK001’ showed a metabolic profile containing 1 amino acid, 2 fatty acids, 5 flavonoids, 5 phenols and avenacoside A in the leaves, and 3 amino acids, 1 carboxylic acid, 1 fatty acid, 1 flavonoid, 4 phenols and avenacin A-1 in the roots. Based on these differential metabolic profiles, clear overlap and differences can be seen among the cultivars in the form of a Venn diagram (Figure 8).

**Figure 9:**
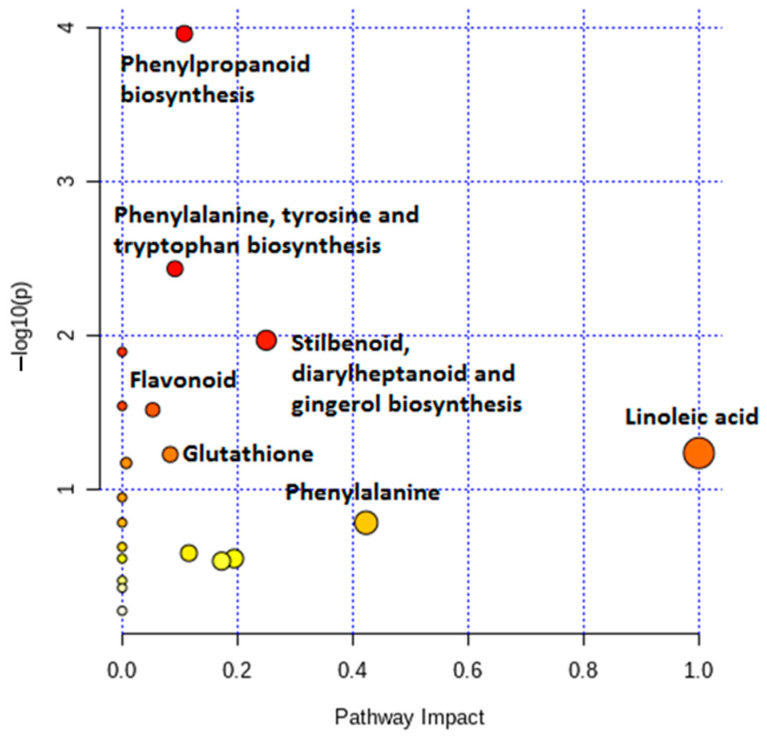
Summarised pathway analyses of all MetaboAnalyst-computed metabolic pathways displayed according to their significance or pathway impact. The figure illustrates all the matched pathways arranged by *p*-values (*y*-axis; pathway enrichment analysis) and the pathway impact values (*x*-axis; pathway topology analysis). Each node is coloured according to its corresponding *p*-values, with the node sizes determined according to their impact values. The graph thus illustrates the pathways with high impact: linoleic acid (C18:2, n-6) pathway, phenylalanine and stilbenoid biosynthesis, and the pathways with high statistical significance: phenylpropanoid, phenylalanine, tyrosine and tryptophan biosynthesis.

Metabolic pathway analyses were performed using the chemometrically extracted metabolites and revealed significant and impactful metabolic pathways. The relative intensities of the different metabolites are illustrated via pie charts among the different pathways. Additionally, colour-coded PCA score plots were used to visually display the presence and abundance of selected discriminant metabolites among the different cultivars (expressed as integrated peak areas in the X data matrix) using vector continuous properties available in SIMCA software (Supplementary Figure S2). The most significant pathways included: aromatic amino acid (Phe, Tyr and Trp) biosynthesis, the phenylpropanoid and flavonoid pathways and the stilbenoid biosynthesis pathway. The linoleic acid pathway was illustrated to be most impactful, followed by phenylalanine (Figure 9). Phenylpropanoid metabolic pathways involve some of the most widely occurring plant secondary metabolites which exhibit a range of biological functions involved in development, defence against biotic and abiotic stresses and modulation of biochemical processes. Additionally, phenylpropanoids are also important for the biosynthesis of key compounds such as flavonoids, coumarins and lignans [37]. Linoleic acids (C18:2) are unsaturated fatty acids that are abundant in plant membranes, important for plant cell structure and maintaining water permeability. Additional desaturation leads to linolenic acid, a precursor molecule in the synthesis of jasmonates, which act as signalling molecules in response to tissue damage caused by pathogens, insects, herbivores or mechanical stress [38].

**Figure 10:**
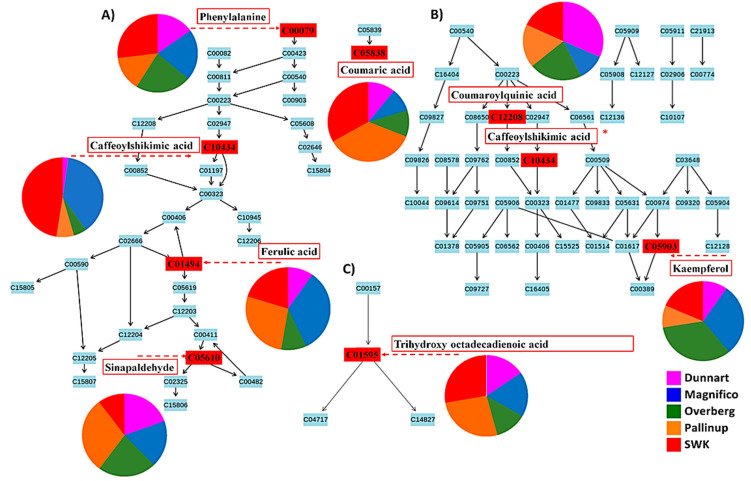
Pathways flagged from metabolomics analysis using MetaboAnalyst software. Signatory metabolites involved in each pathway are illustrated in the form of a pie chart according to their relative intensities and presence across the different cultivars. (**A**) Phenylpropanoid pathway, (**B**) flavonoid pathway overlapping with the phenylpropanoid pathway (*) and (**C**) linoleic acid pathway that showed the highest impact after pathway enrichment analysis. Some limitations in MetaboAnalyst prevented the mapping of all annotated metabolites (Table 1). The respective metabolite codes (e.g., C00079-Phenylalanine) indicate KEGG unique identifiers.

**Figure 11:**
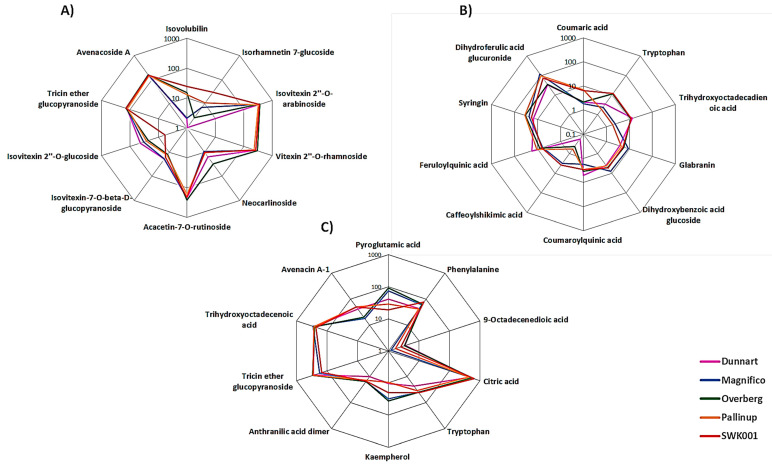
Radar charts illustrating relative intensities of the respective metabolites across the different cultivars in leaf (**A**,**B**) and root (**C**) tissue. The relative peak intensities were averaged and illustrated as log-transformed values.

Differences with regard to the relative intensities of putative signatory biomarkers among the cultivars were thus explored using heatmaps (Figure 6 and Figure 7), colour-coded PCA score plots (Supplementary Figure S2), pie charts (Figure 10) and radar charts (Figure 11). Among the cultivars, ‘Pallinup’ extracts contained a number of metabolites that uniquely presented as discriminatory for this cultivar (hydroxyoctadecatrienoic acid, sinapic acid glucoside, oxalate derivative and isoquercetin in leaves, and trihydroxyoctadecadienoic acid in roots). However, this does not necessarily always indicate absence in the other cultivars but only a greater relative intensity, as can be seen in the generated heatmap (Figure 6). ‘Overberg’ had two uncommon flavonoids that presented as discriminatory for this cultivar: xeractinol (a flavanol C-glucoside) and neocarlinoside (a tetrahydroxyflavone C-glycoside), as can be seen in the illustrated heatmap (Figure 6). The radar chart (Figure 11A) reiterates neocarlinoside as discriminatory in ‘Overberg’ based on its intensity.

Caffeoylshikimic acid is another metabolite that was detected as discriminatory for ‘Magnifico’ and ‘SWK001’. The Figure 6 heatmap illustrates the presence of caffeoylshikimic acid among these cultivars and demonstrates the abundance to be greater in the ‘SWK001’ cultivar compared to all the other cultivars, followed by ‘Magnifico’. This information is further confirmed by Figure 10A (pie chart), illustrating the pathways in which this metabolite is involved and how it is distributed amongst the cultivars, Figure 11B (radar chart) and Figure S2 (colour-coded PCA score plot). ‘Dunnart’ showed a greater abundance of feruloylquinic acid in both leaf and root tissue (Figure 6 and Figure 7), thus making this compound a discriminatory ion for this cultivar. Based on these examples, it is clear that this method of cultivar profiling is sensitive enough to detect the presence of specific secondary metabolites among the different cultivars and generate relative intensity values, thus making it useful in cultivar profiling and comparison.

The averaged peak intensities of each metabolite were also combined to produce radar charts (Figure 11), comparatively displaying features from the metabolomes of the leaf and root tissues. A radar chart is a graphical method used to display multivariate data in a two-dimensional plane and illustrates several quantitative variables on axes originating from the same point. These charts are informative as they sort the variables into different positions that show distinct correlations between the different groups [39]. In the respective radar plots, a range of metabolites are presented and plotted based on their averaged peak intensities. In Figure 11A, clear differences and correlations can be seen. Isovitexin 2″-*O*-glucoside showed to be least abundant in ‘SWK001’ and most abundant in ‘Dunnart’. Isorhamnetin glucoside, on the other hand, was demonstrated as least abundant in ‘Dunnart’ and most abundant in ‘SWK001’. These charts are therefore informative in distinguishing between the various cultivars based on the respective discriminatory metabolites.

**Figure 12:**
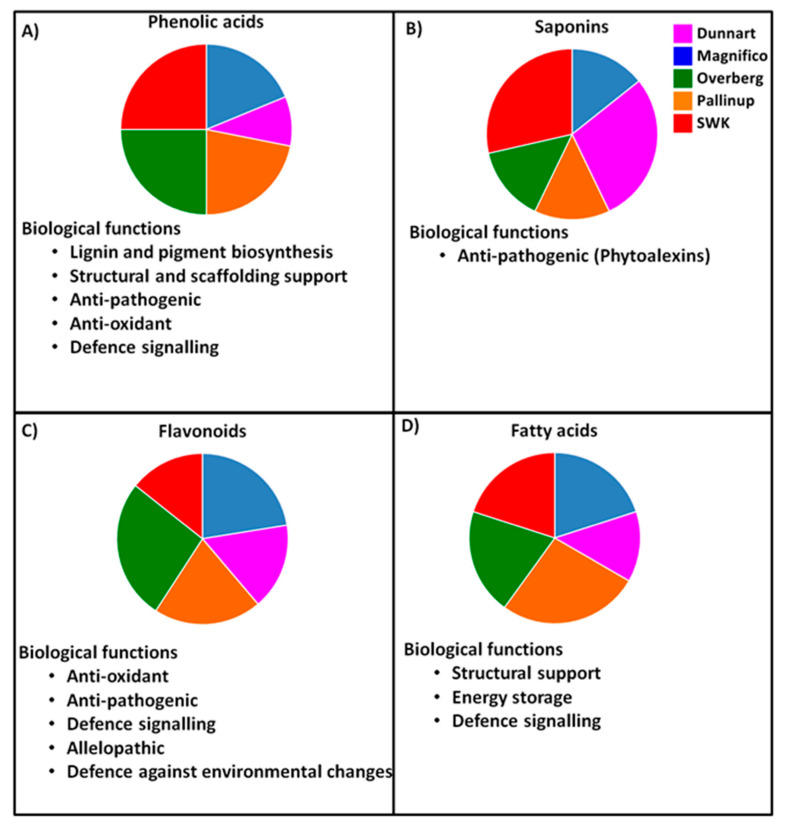
The distribution of the identified metabolite classes across the respective cultivars (Overberg, Pallinup, Dunnart, Magnifico and SWK001). This figure summarises cultivar-related differences due to underlying metabolic profiles of both leaves and roots. Each pie chart illustrates where a greater number of discriminatory metabolites were identified for each class across the cultivars. (**A**) Phenolic acids: discriminatory for the ‘SWK001’ and ‘Overberg’ cultivars. (**B**) Saponins (avenacoside A and avenacin A-1): discriminatory for ‘SWK001’ and ‘Dunnart’. (**C**) Flavonoids: discriminatory for ‘Magnifico’ and ‘Overberg’. (**D**) Fatty acids: discriminatory for ‘Pallinup’.

To summarise, the results show clear cultivar-related differences with regard to the respective underlying metabolic profiles. Metabolomics as a tool for cultivar discrimination would thus provide a quicker view of the metabolome that could be applied in plant breeding studies to not only differentiate but also elucidate possible predictive stress-associated resistance or susceptibility traits among the cultivars. The results show metabolic differences for carboxylic acids, amino acids, fatty acids, phenolic acids (hydroxycinnamic acids and hydroxybenzoic acids and associated derivatives), flavonoids and saponins. Figure 12 summarises the distribution of the discriminatory metabolic markers from the respective metabolite classes (represented are phenolic acids, saponins, flavonoids and fatty acids) and their associated biological functions.

Based on the graphical summary, clear differences and overlap can be seen among the different cultivars. For instance, ‘Overberg’ contains a greater number of phenolics and flavonoids as discriminatory markers; therefore, this cultivar could exhibit a multitude of beneficial traits related to the presence of metabolites from these classes such as antioxidant and antipathogenic activity. The ‘Dunnart’ and ‘SWK001’ cultivars can be seen as containing both avenacoside A and avenacin A-1 as discriminatory, which could suggest comparative greater defence-associated capabilities in leaves and roots based on the biological activity of these compounds. Ultimately, these metabolic features and their differences contribute to biological variances and could affect how the respective cultivars respond to abiotic and biotic factors.

## 3. Discussion

When compared to other cereal crops, oat has been greatly underrated, despite containing a range of unique compounds and nutrients that are greatly beneficial for human health and reduce incidences of certain degenerative diseases [40]. Oat is also considered superior due to its hardiness and ability to thrive and withstand environmentally poor conditions where other cereals seem to be lacking [25]. These benefits are greatly attributed to the rich diversity of secondary metabolites that oat contains such as phenolic acids, flavonoids, phytosterols, carotenoids, avenanthramides, avenacosides and avenacins [41,42]. Among the groups of metabolites identified, carboxylic acids are widely distributed in nature and involved in primary metabolism, responsible for growth and development [43]. For example, citric acid was annotated among the discriminatory ions present in the root extracts. This primary metabolite forms part of the tricarboxylic acid (TCA) cycle and is a pivotal part of energy synthesis, and it provides precursors for the biosynthesis of a range of secondary metabolites and amino acids in plants [44]. Its detection by OPLS-DA and its presence are therefore important in interpreting the metabolic processes that occur and its role in the synthesis of secondary metabolites that were detected as discriminatory metabolites in the respective cultivars.

Common substrates for the synthesis of amino acids include not only intermediates from the TCA cycle but also glycolysis and the pentose phosphate pathway. The latter primarily produces intermediates involved in the synthesis of Phe, Tyr and Trp [45]. Among these, phenylalanine and tryptophan presented as discriminatory metabolites in the leaves and roots. Tryptophan is involved in two distinct pathways to produce secondary metabolites. One such pathway starts with the decarboxylation of tryptophan by tryptophan decarboxylase (TDC) to initiate the synthesis of indole alkaloids. The tryptophan pathway also branches from the shikimate pathway at chorismate, where it is initially synthesised from anthranilate (another discriminatory metabolite) by anthranilate synthase. The secondary metabolites produced via these pathways have been known to play pivotal roles in the defence systems in various members of the grass family (*Poaceae*) [46,47]. Anthranilate also plays an important role in the synthesis of avenanthramides (Ava), which are oat phytoalexins that are produced in response to pathogen infection. Ava have been found to form dimers and are incorporated into plant cell walls for reinforcement; thus, they function in both the chemical and physical defence of oat against pathogens [48,49].

Flavonoids are synthesised through the phenylpropanoid pathway (Figure 10A), where 4-coumaroyl-CoA is formed from cinnamic acid, which finally enters the flavonoid (Figure 10B) biosynthesis pathway [50]. The first enzyme specific for the flavonoid pathway, chalcone synthase, produces chalcone scaffolds from which all flavonoids derive. Flavonoids most common in oat include apigenin, luteolin, tricin, kaempferol, quercetin and their glycoside derivatives [51,52]. The majority of metabolites identified were classified as flavonoids, with most being glycoside derivatives of apigenin, quercetin, kaempferol and tricin. Flavonoids have a range of biological activities in plants such as antioxidant, antimicrobial, signalling, allelopathic and defence against environmental stressors [53].

Phenolic acids are synthesised via the phenylpropanoid pathway from phenylalanine through a process that commonly involves deamination, hydroxylation and methylation [54]. Structurally, all phenolic acids are hydroxylated derivatives of cinnamic acid or benzoic acid. Hydroxycinnamic acid (HCA) derivatives commonly include ferulic acid, caffeic acid, sinapic acid and coumaric acid. Correspondingly, hydroxybenzoic acids include derivatives known as protocatechuic acid, gallic acid, vanillic acid and sinapinic acid [55]. HCAs were abundantly identified among the various cultivars, with derivatives from coumaric acids, ferulic acid and sinapic acid commonly present. These phenolics are generally known to be significant in plant development, particularly in lignin and pigment biosynthesis, and provide structural and scaffolding support to plants [56].

Plants also produce a range of fatty acids, some of which presented as discriminatory metabolites among the cultivars. Commonly, plants produce palmitic, oleic, linoleic and linolenic acids, and in this study, oleic acid derivatives were frequently identified among the cultivars. Oleic acid is converted to linoleic acid which, in turn, is converted to linolenic acid [57]. Oleic and linoleic acids are known to constitute the two major unsaturated fatty acids in plants and are involved in a range of biological activities, some of which include antifungal properties, and also in the synthesis of important defence signalling molecules such as jasmonates [58,59].

Two saponin molecules were also identified in the leaves (avenacoside A) and the roots (avenacin A-1) of the respective cultivars. Avenacosides are biologically inactive phytoanticipins that are converted into biologically active 26-desglucoavenacosides by an avenacosidase enzyme in response to tissue damage or pathogen attack [60]. The major mechanism of activity against pathogens is due to their ability to complex with sterols in the pathogen membrane and cause disruption in the membrane integrity. This process is thought to result in the formation of transmembrane pores by aggregation of the saponin with the sterol groups. The remaining sugar moieties of the active molecules have also been known to play an essential role in membrane permeabilisation, and therefore the removal of these sugar residues could result in loss of biological activity [60,61,62,63]. Avenacins have a similar mechanism of action against pathogens that attack the roots; however, they are already present in biologically active forms. Ultimately, these saponins are responsible for defence against pathogens via the formation of micelle-like aggregations between the saponins and sterols in the membrane [64,65].

## 4. Materials and Methods

### 4.1. Plant Cultivation

Seeds of five oat (*Avena sativa* L.) cultivars: ‘Magnifico’, ‘Dunnart’, ‘Pallinup’, ‘Overberg’ (Agricol, Pretoria, South Africa) and ‘SWK001’ (ARC Small Grain Institute, Bethlehem, South Africa), were obtained and cultivated in triplicate. All cultivars were grown in germination mixture (Culterra, Muldersdrift, South Africa) under greenhouse conditions: a light/dark cycle of 12 h/12 h, with a light intensity of about 84 µmol/m^2^/s and temperature between 25 and 28 °C. Once the plants reached the 3-week maturity stage (seedling stage or three-leaf stage), the leaves and roots were harvested, frozen in liquid nitrogen to quench metabolic activity and stored at −80 °C until metabolite extraction. The experimental design included three independent biological replicates and the experiments were repeated twice.

### 4.2. Metabolite Extraction and Sample Preparation

Liquid nitrogen was added to the leaf and root materials, which were then crushed into powder form using a mortar and pestle. One gram per sample was weighed into a clean 50 mL Falcon tube and 10 mL of 80% cold aqueous methanol (4 °C) was added (*m*/*v* ratio of 1:10). The methanol used was analytical grade (Rochelle Chemicals, Johannesburg, South Africa). The mixture was then homogenised using a probe sonicator (Bandelin Sonopuls, Berlin, Germany) set to 55% power for 10 s per sample. Equipment was cleaned between samples to prevent cross-contamination. The homogenates were centrifuged at 5100× *g* for 20 min at 4 °C in a benchtop centrifuge after which the supernatants were kept and concentrated by evaporating the methanol under vacuum to approximately 1 mL using a rotary evaporator set to 55 °C. The concentrated samples were transferred to 2 mL Eppendorf microcentrifuge tubes and dried in a centrifugal evaporator under vacuum. The dried extracts were then reconstituted by dissolving in 500 μL of 50% aqueous methanol (LC-grade, Romil Pure Chemistry, Cambridge, UK). The samples were subsequently filtered through nylon syringe filters (0.22 μm) into chromatography vials fitted with 500 μL inserts, capped and kept at 4 °C until analysis.

### 4.3. Ultra-High Performance Liquid Chromatography (UHPLC) Analyses

An Acquity UHPLC system (Waters Corporation, Manchester, UK) was used to analyse 2 µL of each sample, separated into its respective components using a binary solvent on an HSS T3 reverse-phase column (2.1 × 150 mm × 1.7 µm; Waters Corporation, Billerica, MA, USA). The solvents used were MilliQ water (solvent A) and acetonitrile (solvent B) (Romil Chemistry, Cambridge, UK), both containing 0.1% formic acid (Sigma, Munich, Germany) and 2.5% isopropanol (IPA, Romil, Cambridge, UK). The run was set to 30 min per 2 μL injection with an elution gradient carried out via a binary solvent system at a flow rate of 0.4 mL/min. The initial conditions were 95% A and 5% B held for 1 min. A gradient was applied to change the chromatographic conditions to 10% A and 90% B at 25 min and changed to 5% A and 95% B at 25.10 min. These conditions were held for 2 min and then changed to the initial conditions at 28 min. The analytical column was allowed to calibrate for 2 min before the next injection. Pooled quality control (QC) samples were also prepared to condition the LC–MS system and assess the reliability and reproducibility of each analysis [66]. Additionally, blank samples (50% MeOH) were also randomly included in the run to monitor potential carry over and background noise. Each sample was analysed in triplicate (technical replicates), and together with the three biological replicates, this generated n = 9, in order to account for analytical variability.

### 4.4. Quadrupole Time-of-Flight Mass Spectrometry (q–TOF–MS)

A high-definition SYNAPT G1 q-TOF mass spectrometry system, controlled by MassLynx XS^TM^ software (Waters Corporation, Manchester, UK), was coupled to the chromatography system to detect metabolites and acquire data in both positive and negative electrospray ionisation (ESI) operation modes. A reference calibrant, leucine encephalin (554.2615 Da), was set as the lockmass and allowed for typical mass accuracies from 1 to 3 mDa. The respective capillary and sampling cone voltages were set as 2.5 kV and 30 V. The desolvation temperature used was 450 °C, with the source temperature set to 120 °C, cone gas flow set to 50 L/h and desolvation gas flow set to 550 L/h. An *m*/*z* range of 50–1200 Da was set with a scan time of 0.1 s. The desolvation, collision and cone gas used at a flow rate of 700 L/h was high-purity nitrogen. Data were acquired using five different collision energies (MS^E^), ramping from 0 to 50 eV to cause fragmentation of the initial ions so as to ensure that as much information regarding the structures of the respective compounds could be obtained for downstream structural elucidation and metabolite annotation [67,68].

### 4.5. Data Analyses

The datasets obtained were explored and processed using MarkerLynx XS^TM^ software (Waters Corporation, Manchester, UK). The software makes use of a patented algorithm called ApexTrack. The following parameters were used for processing: retention time (Rt) range 2–25 min and *m/z* range 150–1200 Da. The Rt window was set to 0.20 min and the mass window to 0.05 Da. The mass tolerance was 0.05 Da and the intensity threshold was set to 150 counts. The generated data matrices were exported into “soft independent modelling of class analogy” (SIMCA) software, version 14 (Umetrics, Umea, Sweden), for multivariate data analysis (MVDA). Unsupervised models, namely, principal component analysis (PCA) and hierarchical clustering analysis (HiCA), were used to reduce the dimensionality of the datasets and to explore the underlying structures and characteristics of the data. Supervised orthogonal projection to latent structures discriminant analysis (OPLS-DA) was used for binary classification analyses of cultivars, identifying thus discriminatory ions among the different cultivars. The OPLS-DA models were validated using rigorous methods [10,69,70]. The roles of these MDVA tools in the metabolomics workflow are further described in Section 2.2.

### 4.6. Metabolite Annotation and Semi-Quantitative Comparisons

Metabolites were putatively identified based on their respective (measured) accurate masses (based on which elemental compositions were computed using the MarkerLynx XS software tool) and fragmentation information (for structural elucidation). Each suggested empirical formula was exported and searched for in various databases such as MetaCyc [71], Plant Metabolic Network (PMN) [72], ChemSpider, MassBank of North America [73], Dictionary of Natural Products [74] and the Kyoto Encyclopedia of Genes and Genomes (KEGG) [75]. Processed data matrices were also exported from MarkerLynx XS software to the “Taverna workbench” containing an in-house library and allowing a high-throughput automated assignment of putative metabolite identities based on measured accurate masses and other collected spectral features (for a detailed description, see [76]. Metabolites were putatively identified to level 2 of the Metabolomics Standards Initiative (MSI) unless specified otherwise [77]. Avenacoside A was identified using an authentic standard (Sigma-Aldrich, Muenchen, Germany).

Furthermore, MetaboAnalyst 4.0 https://www.metaboanalyst.ca/home.xhtml (accessed on 12 March 2021) [36] was utilised for additional integrative data analyses. Data pre-treatment (integrity, missing values, filtering and normalisation) was performed prior to downstream chemometric and statistical modelling. A comparison of the magnitude and presence of the identified metabolites among the various cultivars was performed via heatmap analyses using a Pearson distance measure and the Ward clustering algorithm [36,78]. Partial least square discriminant analysis (PLS-DA) was also used to mine the data via MetaboAnalyst for the comparison and visualisation of the relative abundances of the identified metabolites across the various cultivars. “Variable importance in projection” (VIP) score plots, derived from the OPLS-DAs, were generated to indicate the key discriminatory metabolites with VIP scores of >0.5 which are considered significant in discriminating between the cultivars. Additionally, to further visualise changes among the discriminatory metabolites across the various cultivars, radar plots were constructed based on the averages of the relative intensities and illustrated as log-transformed values (Section 2.3).

## 5. Conclusions

Metabolomics has been widely applied in crop plant sciences and has shown great progress in understanding how the phenotype links to the metabolome and, by extension, elucidating the active role of metabolites under normal and stress conditions. Metabolomics could therefore provide insights into understanding crop physiology and biochemistry as well as underlying metabolic events. This could greatly improve crop breeding which is currently based on gene and marker-assisted selection. Although the latter has shown success in crop improvement, it is also faced with many limitations such as the fact that the presence of a gene does not necessarily ensure the expression of a trait. Metabolomics has the potential to overcome this limitation and provide useful insights about metabolites involved in resistance, growth and stress responses, which, in turn, can be applied to crop improvement. Thus, in this study, LC–MS-based metabolomics was applied to interrogate the metabolomes of five different oat cultivars. This multidisciplinary omics approach allowed the elucidation and characterisation of differential metabolic profiles that define natural variation among the oat metabolomes under consideration. The identified metabolic classes were carboxylic acids, amino acids, fatty acids, phenolic compounds (hydroxycinnamic acids and hydroxybenzoic acids and associated derivatives) and flavonoids. Further, a steroidal saponin (avenacoside A) was annotated in extracts from leaves and a triterpenoid saponin (avenacin A-1) was annotated in extracts from roots. The differences in the metabolic profiles indicate that untargeted metabolomics can be used to distinguish between cultivars. The results further indicate that to discriminate between the different cultivars, the presence or absence of specific metabolites cannot be the only concluding factor, and the relative intensities or ratios of the metabolites also need to be considered as distinguishing criteria. The secondary metabolite classes that were mentioned have various biological roles that are important in plant growth and development, preventing pathogen infections and maintaining the plant under various environmental conditions. Ultimately, an untargeted LC–MS-based metabolomics approach can be used to detect the underlying metabolites that contribute to phenotypic and physiological traits. This will greatly contribute to a more holistic comprehension of the oat plant metabolome which can ultimately be applied in crop improvement and breeding strategies.

## Figures and Tables

**Table 1:** List of key discriminatory metabolites putatively identified from leaves and roots of the oat cultivars ‘Magnifico’ (Mag), ‘Dunnart’ (Dun), ‘Pallinup’ (Pal), ‘Overberg’ (Over) and ‘SWK001’ (SWK). These metabolites were identified based on OPLS-DA S-plots, with a rigorous statistical validation. All metabolites had variable importance in projection (VIP) scores >1.0.

Annotated Metabolites	Molecular Formula	ESI Mode	*m/z*	Rt (min)	Metabolite Class	Cultivars				
						‘Mag’	‘Dun’	‘Pal’	‘Over’	‘SWK’
**Leaves**										
Coumaric acid	C_9_H_8_O_3_	Neg	163.0379	3.35	Phenolic acid			*		*
Tryptophan	C_11_H_12_N_2_O_2_	Neg	203.081	2.48	Amino acid				*	*
Hydroxyoctadecatrienoic acid	C_18_H_30_O_3_	Neg	293.208	21.49	Fatty acid			*		
Dihydroxybenzoic acid glucoside	C_13_H_16_O_9_	Neg	315.0742	2.52	Phenolic acid	*				*
Glabranin	C_20_H_20_O_4_	Neg	323.1326	2.94	Flavonoid	*			*	
Trihydroxyoctadecadienoic acid	C_18_H_32_O_5_	Neg	327.2153	16.63	Fatty acid			*		*
Trihydroxyoctadecenoic acid	C_18_H_34_O_5_	Neg	329.23	17.34	Fatty acid			*		*
Caffeoylshikimic acid	C_16_H_16_O_8_	Neg	335.0422	2.24	Phenolic acid	*				*
Coumaroylquinic acid	C_16_H_18_O_8_	Neg	337.092	3.19	Phenolic acid		*		*	
Sinapoylglutamine	C_16_H_20_N_2_O_7_	Neg	351.1257	6.48	Phenolic acid	*				
Feruloylquinic acid	C_17_H_20_O_9_	Neg	367.1008	4.01	Phenolic acid		*			
Sinapaldehyde glucoside	C_17_H_22_O_9_	Neg	369.1184	13.54	Phenolic acid		*	*	*	*
Dihydroferulic acid glucuronide	C_16_H_20_O_10_	Neg	371.0958	7.21	Phenolic acid	*		*		*
Syringin	C_17_H_24_O_9_	Neg	371.1346	16.0	Phenolic acid	*		*	*	
Sinapic acid glucoside	C_17_H_22_O_10_	Neg	385.1146	3.40	Phenolic acid			*		
Auriculoside	C_22_H_26_O_10_	Neg	393.1752	12.10	Flavonoid	*		*		*
Nobiletin	C_21_H_22_O_8_	Pos	403.1454	9.53	Flavonoid			*	*	
Sophoraflavanone G	C_25_H_28_O_6_	Neg	423.1856	11.83	Flavonoid	*		*	*	
Licoricidin	C_26_H_32_O_5_	Neg	423.2204	14.81	Flavonoid	*	*			*
Isovolubilin	C_23_H_24_O_9_	Neg	443.1328	16.81	Flavonoid			*	*	*
Isoquercetin	C_21_H_20_O_12_	Neg	463.0895	6.59	Flavonoid			*		
Xeractinol	C_21_H_22_O_12_	Neg	465.1028	12.97	Flavonoid				*	
Isorhamnetin 7-glucoside	C_22_H_22_O_12_	Neg	477.1038	9.03	Flavonoid	*		*		*
Oxalate derivative	C_25_H_24_O_10_	Neg	483.1281	12.20	Phenolic acid			*		
Isovitexin 2″-*O*-arabinoside	C_26_H_28_O_14_	Pos	563.1393	10.08	Flavonoid				*	*
Vitexin 2″-*O*-rhamnoside	C_27_H_30_O_14_	Neg	577.1545	10.49	Flavonoid	*	*		*	
Neocarlinoside	C_26_H_28_O_15_	Neg	579.1349	8.53	Flavonoid				*	
Acacetin-7-*O*-rutinoside	C_28_H_32_O_14_	Pos	593.186	11.20	Flavonoid	*	*		*	
Isovitexin 2″-*O*-glucoside	C_27_H_30_O_15_	Neg	593.1488	9.94	Flavonoid	*	*	*	*	
Isovitexin-7-*O*-glucopyranoside	C_27_H_30_O_15_	Pos	595.1499	8.50	Flavonoid		*	*		
Prenylkaempferol diglucoside	C_32_H_38_O_16_	Neg	677.207	14.41	Flavonoid	*	*			
Tricin ether glucopyranoside	C_33_H_36_O_16_	Pos	689.194	13.51	Flavonoid			*	*	*
Avenacoside A	C_51_H_82_O_23_	Pos	1063.539	16.58	Triterpene	*	*		*	*
**Roots**										
Pyroglutamic acid	C_5_H_7_NO_3_	Neg	128.033	1.16	Amino acid	*	*		*	
Phenylalanine	C_9_H_11_NO_2_	Neg	164.068	1.67	Amino acid	*			*	*
Citric acid	C_6_H_8_O_7_	Neg	191.0163	1.16	Carboxylic acid	*	*		*	*
Tryptophan	C_11_H_12_N_2_O_2_	Neg	203.081	2.49	Amino acid	*		*	*	*
Anthranilic acid dimer	C_14_H_12_N_2_O_4_	Neg	271.07	2.49	Phenolic acid			*	*	
Kaempferol	C_15_H_10_O_6_	Neg	285.039	12.80	Flavonoid	*			*	*
Hydroxylinoleic acid	C_18_H_32_O_3_	Neg	295.15	23.87	Fatty acid	*			*	*
Octadecenedioic acid	C_18_H_32_O_4_	Neg	311.165	22.78	Fatty acid	*	*		*	
Dihydroxybenzoic acid glucoside	C_13_H_16_O_9_	Neg	315.069	1.67	Phenolic acid				*	*
Hydroxycoumarin glucoside	C_15_H_16_O_8_	Neg	323.097	1.68	Phenolic acid	*			*	*
Trihydroxyoctadecadienoic acid	C_18_H_32_O_5_	Neg	327.214	16.63	Fatty acid			*		
Trihydroxyoctadecenoic acid	C_18_H_34_O_5_	Neg	329.23	17.36	Fatty acid	*	*		*	
Feruloylquinic acid	C_17_H_19_O_9_	Neg	367.101	4.02	Phenolic acid				*	*
Sophoraflavanone G	C_25_H_28_O_6_	Neg	423.186	7.65	Flavonoid		*			
di-Sinapoylglucoside	C_28_H_32_O_14_	Neg	591.1693	11.20	Phenolic acid		*		*	
Tricin ether glucopyranoside	C_33_H_36_O_16_	Neg	687.192	13.06	Flavonoid	*	*	*	*	
Avenacin A-1	C_55_H_83_NO_21_	Neg	1092.55	18.49	Triterpene		*	*		*

The asterisks within the coloured cells indicate metabolites that were discriminatory for the respective cultivars compared to the other four cultivars and are indicated when detected against one or more of the other cultivars.

## Data Availability

The study design information, LC–MS data, data processing and analyses are reported on and incorporated into the main text. Raw data, analyses and data processing information and the meta-data have been deposited into the EMBL-EBI metabolomics repository—MetaboLights50, with the identifier MTBLS2478 (http://www.ebi.ac.uk/metabolights/MTBLS2478) (accessed on 12 March 2021).

## Supplementary Materials

The following are available online at https://www.mdpi.com/2218-1989/11/3/165/s1, Figure S1: An orthogonal projection to latent structures discriminant analysis (OPLS-DA) model of two representative oat cultivars, ‘Dunnart’ and ‘SWK001’. Figure S2: Colour-coded PCA score plots showing the presence and increasing abundance of discriminatory ions in the respective cultivars ‘Overberg’, ‘Pallinup’,’Dunnart’, ‘Magnifico’ and ‘SWK001’.

## References

[1] Tilman D., Balzer C., Hill J., Befort B.L. (2011). Global food demand and the sustainable intensification of agriculture. Proc. Natl. Acad. Sci. USA.

[2] Hundleby P.A.C., Harwood W.A. (2019). Impacts of the EU GMO regulatory framework for plant genome editing. Food Energy Secur..

[3] Korir N.K., Han J., Shangguan L., Wang C., Kayesh E., Zhang Y., Fang J. (2012). Plant variety and cultivar identification: Advances and prospects. Crit. Rev. Biotechnol..

[4] Edwards D., Batley J., Snowdon R.J. (2013). Accessing complex crop genomes with next-generation sequencing. Theor. Appl. Genet..

[5] Boopathi N.M. (2020). Marker-assisted selection (MAS). Genetic Mapping and Marker Assisted Selection.

[6] Yandeau-Nelson M.D., Lauter N., Zabotina O.A. (2015). Advances in metabolomic applications in plant genetics and breeding. CAB Rev..

[7] Gienapp P., Laine V.N., Mateman A.C., van Oers K., Visser M.E. (2017). Environment-dependent genotype-phenotype associations in avian breeding time. Front. Genet..

[8] Rosato A., Tenori L., Cascante M., Carulla P.R.D.A., Dos Santos V.A.P.M., Saccenti E. (2018). From correlation to causation: Analysis of metabolomics data using systems biology approaches. Metabolomics.

[9] Handakumbura P.P., Stanfill B., Rivas-Ubach A., Fortin D., Vogel J.P., Jansson C. (2019). Metabotyping as a stopover in genome-to-phenome mapping. Sci. Rep..

[10] Hamany Djande C.Y., Pretorius C., Tugizimana F., Piater L.A., Dubery I.A. (2020). Metabolomics: A tool for cultivar phenotyping and investigation of grain crops. J. Agron..

[11] Tugizimana F., Piater L., Dubery I. (2013). Plant metabolomics: A new frontier in phytochemical analysis. S. Afr. J. Sci..

[12] Kapoore R.V., Vaidyanathan S. (2016). Towards quantitative mass spectrometry-based metabolomics in microbial and mammalian systems. Philos. Trans. R. Soc. A.

[13] Perez E.R., Knapp J.A., Horn C.K., Stillman S.L., Evans J.E., Arfsten D.P. (2016). Comparison of LC–MS-MS and GC–MS Analysis of Benzodiazepine Compounds Included in the Drug Demand Reduction Urinalysis Program. J. Anal. Toxicol..

[14] Shimizu T., Watanabe M., Fernie A.R., Tohge T. (2018). Targeted LC-MS analysis for plant secondary metabolites. Plant Metabolomics.

[15] Gathungu R.M., Kautz R., Kristal B.S., Bird S.S., Vouros P. (2020). The integration of LC-MS and NMR for the analysis of low molecular weight trace analytes in complex matrices. Mass Spectrom. Rev..

[16] Kumar R., Bohra A., Pandey A.K., Pandey M.K., Kumar A. (2017). Metabolomics for Plant Improvement: Status and Prospects. Front. Plant Sci..

[17] Razzaq A., Sadia B., Raza A., Hameed M.K., Saleem F. (2019). Metabolomics: A Way Forward for Crop Improvement. Metabolites.

[18] Ivanisevic J., Want E.J. (2019). From Samples to Insights into Metabolism: Uncovering Biologically Relevant Information in LC-HRMS Metabolomics Data. Metabolites.

[19] Hussein R.A., El-Anssary A.A., Builders P.H. (2018). Plants secondary metabolites: The Key drivers of the pharmacological actions of medicinal plants. Herbal Medicine.

[20] Isah T. (2019). Stress and defense responses in plant secondary metabolites production. Biol. Res..

[21] Steinfath M., Strehmel N., Peters R., Schauer N., Groth D., Hummel J., Steup M., Selbig J., Kopka J., Geigenberger P. (2010). Discovering plant metabolic biomarkers for phenotype prediction using an untargeted approach. Plant Biotechnol. J..

[22] Peng B., Li H., Peng X.-X. (2015). Functional metabolomics: From biomarker discovery to metabolome reprogramming. Protein Cell.

[23] Sarwar M.H., Sarwar M.F., Sarwar M., Qadri N.A., Moghal S. (2013). The importance of cereals (*Poaceae: Gramineae*) nutrition in human health: A review. J. Cereals Oilseeds.

[24] Jing P., Hu X., Yu L., Tsao R., Shahidi F. (2012). Nutraceutical properties and health benefits of oats. Cereals and Pulses: Nutraceutical Properties and Health Benefits.

[25] Rasane P., Jha A., Sabikhi L., Kumar A., Unnikrishnan V.S. (2013). Nutritional advantages of oats and opportunities for its processing as value added foods—A review. J. Food Sci. Technol..

[26] Saini P., Gani M., Saini P., Bhat J.A., Francies R.M., Negi N., Chauhan S.S., Wani S.H. (2019). Molecular breeding for resistance to economically important diseases of fodder oat. Disease Resistance in Crop Plants.

[27] Boccard J., Rudaz S. (2014). Harnessing the complexity of metabolomic data with chemometrics. J. Chemom..

[28] Brereton R.G. (2015). Pattern recognition in chemometrics. Chemom. Intell. Lab. Syst..

[29] Tebani A., Afonso C., Bekri S. (2018). Advances in metabolome information retrieval: Turning chemistry into biology. Part II: Biological information recovery. J. Inherit. Metab. Dis..

[30] Jolliffe I.T., Cadima J. (2016). Principal component analysis: A review and recent developments. Philos. Trans. R. Soc. A Math. Phys. Eng. Sci..

[31] Handorf E.A., Heckman C.J., Darlow S., Slifker M., Ritterband L. (2018). A hierarchical clustering approach to identify repeated enrollments in web survey data. PLoS ONE.

[32] Lemoine R., La Camera S., Atanassova R., Dédaldéchamp F., Allario T., Pourtau N., Bonnemain J.L., Laloi M., Coutos-Thévenot P., Maurousset L. (2013). Source-to-sink transport of sugar and regulation by environmental factors. Front. Plant Sci..

[33] White A.C., Rogers A., Rees M., Osborne C.P. (2015). How can we make plants grow faster? A source-sink perspective on growth rate. J. Exp. Bot..

[34] Mugford S.T., Osbourn A., Bach T., Rohmer M. (2012). Saponin Synthesis and Function. Isoprenoid Synthesis in Plants and Microorganisms.

[35] Worley B., Powers R. (2016). PCA as a practical indicator of OPLS-DA model reliability. Curr. Metab..

[36] Chong J., Soufan O., Li C., Caraus I., Li S., Bourque G., Wishart D.S., Xia J. (2018). MetaboAnalyst 4.0: Towards more transparent and integrative metabolomics analysis. Nucleic Acids Res..

[37] Fraser C.M., Chapple C. (2011). The Phenylpropanoid Pathway in Arabidopsis. Arab. Book.

[38] Wasternack C., Song S. (2017). Jasmonates: Biosynthesis, metabolism, and signaling by proteins activating and repressing transcription. J. Exp. Bot..

[39] Porter M.M., Niksiar P. (2018). Multidimensional mechanics: Performance mapping of natural biological systems using permutated radar charts. PLoS ONE.

[40] Stewart D., McDougall G. (2014). Oat agriculture, cultivation and breeding targets: Implications for human nutrition and health. Br. J. Nutr..

[41] Belobrajdic D.P., Bird A.R. (2013). The potential role of phytochemicals in wholegrain cereals for the prevention of type-2 diabetes. Nutr. J..

[42] Sang S., Chu Y. (2017). Whole grain oats, more than just a fiber: Role of unique phytochemicals. Mol. Nutr. Food Res..

[43] Badea G.I., Radu G.L. (2018). Introductory chapter: Carboxylic acids—Key role in life sciences. Carboxylic Acid: Key Role in Life Sciences.

[44] Zhang Y., Fernie A.R. (2018). On the role of the tricarboxylic acid cycle in plant productivity. J. Integr. Plant Biol..

[45] Tzin V., Galili G. (2010). New Insights into the Shikimate and Aromatic Amino Acids Biosynthesis Pathways in Plants. Mol. Plant.

[46] Ishihara A., Matsuda F., Miyagawa H., Wakasa K. (2007). Metabolomics for metabolically manipulated plants: Effects of tryptophan overproduction. Metabolomics.

[47] Kokubo Y., Nishizaka M., Ube N., Yabuta Y., Tebayashi S.-I., Ueno K., Taketa S., Ishihara A. (2017). Distribution of the tryptophan pathway-derived defensive secondary metabolites gramine and benzoxazinones in *Poaceae*. Biosci. Biotechnol. Biochem..

[48] Okazaki Y., Isobe T., Iwata Y., Matsukawa T., Matsuda F., Miyagawa H., Ishihara A., Nishioka T., Iwamura H. (2004). Metabolism of avenanthramide phytoalexins in oats. Plant J..

[49] Li Z., Chen Y., Meesapyodsuk D., Qiu X. (2019). The Biosynthetic Pathway of Major Avenanthramides in Oat. Metabolites.

[50] Ferreyra M.L.E., Rius S.P., Ecasati P. (2012). Flavonoids: Biosynthesis, biological functions, and biotechnological applications. Front. Plant Sci..

[51] Peterson D.M. (2001). Oat Antioxidants. J. Cereal Sci..

[52] Krošlák E., Maliar T., Maliarová M., Nemeček P., Hozlár P., Ondrejovič M., Havrlentová M., Kraic J. (2016). Antioxidant and protease-inhibitory potential of extracts from grains of oat. Open Chem..

[53] Panche A.N., Diwan A.D., Chandra S.R. (2016). Flavonoids: An overview. J. Nutr. Sci..

[54] Vogt T. (2010). Phenylpropanoid Biosynthesis. Mol. Plant.

[55] Lafay S., Gil-Izquierdo A. (2007). Bioavailability of phenolic acids. Phytochem. Rev..

[56] Bhattacharya A., Sood P., Citovsky V. (2010). The roles of plant phenolics in defence and communication during Agrobacterium and Rhizobium infection. Mol. Plant Pathol..

[57] Aid F. (2019). Plant lipid metabolism. Advances in Lipid Metabolism.

[58] Walley J.W., Kliebenstein D.J., Bostock R.M., Dehesh K. (2013). Fatty acids and early detection of pathogens. Curr. Opin. Plant Biol..

[59] He M., Qin C.-X., Wang X., Ding N.-Z. (2020). Plant Unsaturated Fatty Acids: Biosynthesis and Regulation. Front. Plant Sci..

[60] Pecio Ł., Wawrzyniak-Szołkowska A., Oleszek W., Stochmal A. (2013). Rapid analysis of avenacosides in grain and husks of oats by UPLC–qTOF–MS. Food Chem..

[61] Morrissey J.P., Wubben J.P., Osbourn A.E. (2000). *Stagonospora avenae* secretes multiple enzymes that hydrolyze oat leaf saponins. Mol. Plant Microbe Interact..

[62] Du Fall L.A., Solomon P.S. (2011). Role of Cereal Secondary Metabolites Involved in Mediating the Outcome of Plant-Pathogen Interactions. Metabolites.

[63] Moses T., Papadopoulou K.K., Osbourn A. (2014). Metabolic and functional diversity of saponins, biosynthetic intermediates and semi-synthetic derivatives. Crit. Rev. Biochem. Mol. Biol..

[64] Armah C., Mackie A., Roy C., Price K., Osbourn A., Bowyer P., Ladha S. (1999). The Membrane-Permeabilizing Effect of Avenacin A-1 Involves the Reorganization of Bilayer Cholesterol. Biophys. J..

[65] Owatworakit A., Townsend B., Louveau T., Jenner H., Rejzek M., Hughes R.K., Saalbach G., Qi X., Bakht S., Roy A.D. (2013). Glycosyltransferases from Oat (Avena) Implicated in the Acylation of Avenacins. J. Biol. Chem..

[66] Broadhurst D., Goodacre R., Reinke S.N., Kuligowski J., Wilson I.D., Lewis M.R., Dunn W.B. (2018). Guidelines and considerations for the use of system suitability and quality control samples in mass spectrometry assays applied in untargeted clinical metabolomic studies. Metabolomics.

[67] Tugizimana F., Steenkamp P.A., Piater L.A., Dubery I.A. (2014). Multi-Platform Metabolomic Analyses of Ergosterol-Induced Dynamic Changes in *Nicotiana tabacum* Cells. PLoS ONE.

[68] Zeiss D.R., Mhlongo M.I., Tugizimana F., Steenkamp P.A., Dubery I.A. (2018). Comparative Metabolic Phenotyping of Tomato (*Solanum lycopersicum*) for the Identification of Metabolic Signatures in Cultivars Differing in Resistance to *Ralstonia solanacearum*. Int. J. Mol. Sci..

[69] Trygg J., Holmes A.E., Lundstedt T. (2007). Chemometrics in Metabonomics. J. Proteome Res..

[70] Worley B., Powers R. (2012). Multivariate Analysis in Metabolomics. Curr. Metab..

[71] MetaCyc. https://metacyc.org/.

[72] PlantCyc. https://plantcyc.org/.

[73] MassBank. https://massbank.eu/MassBank/Search.

[74] Dictionary of Natural Products. www.dnp.chemnetbase.com.

[75] KEGG. http://www.genome.jp/kegg/.

[76] Brown M., Wedge D.C., Goodacre R., Kell D.B., Baker P.N., Kenny L.C., Mamas M.A., Neyses L., Dunn W.B. (2011). Automated workflows for accurate mass-based putative metabolite identification in LC/MS-derived metabolomic datasets. Bioinformatics.

[77] Sumner L.W., Amberg A., Barrett D., Beale M., Beger R., Daykin C.A., Fan T.W.M., Fiehn O., Goodacre R., Griffin J.L. (2007). Proposed minimum reporting standards for chemical analysis. Chemical Analysis Working Group (CAWG). Metabolomics.

[78] Xia J., Psychogios N., Young N., Wishart D.S. (2009). MetaboAnalyst: A web server for metabolomic data analysis and interpretation. Nucleic Acids Res..

