# Supplementary material for: Metabolomics for Biomarker Discovery: Key Signatory Metabolic Profiles for the Identification and Discrimination of Oat Cultivars
Source: Metabolites. 2021 Mar 12;11(3):165. doi: 10.3390/metabo11030165 (PMC8001698; doi:10.3390/metabo11030165)
Supplement: Supplementary file 1 [file metabolites-11-00165-s001.pdf]

Metabolomics for biomarker discovery: Key signatory metabolic profiles for the identification and discrimination of oat cultivars

Chanel J. Pretorius, Fidele Tugizimana, Paul A. Steenkamp, Lizelle A. Piater, Ian A. Dubery*

Research Centre for Plant Metabolomics, Department of Biochemistry, University of Johannesburg, P.O. Box 524, Auckland Park, Johannesburg 2006, South Africa; 201423600@student.uj.ac.za (C.J.P.); ftugizimana@uj.ac.za (F.T.); psteenkamp@uj.ac.za (P.A.S.); lpiater@uj.ac.za (L.A.P.)

* Correspondence: idubery@uj.ac.za; Tel.: +27-11-5592401

Supplementary materials

All relevant material referred to, but not included in the main text are included in the following supplementary file. All experimental raw data is available on request at the Department of Biochemistry, University of Johannesburg with permission from Prof. IA Dubery.

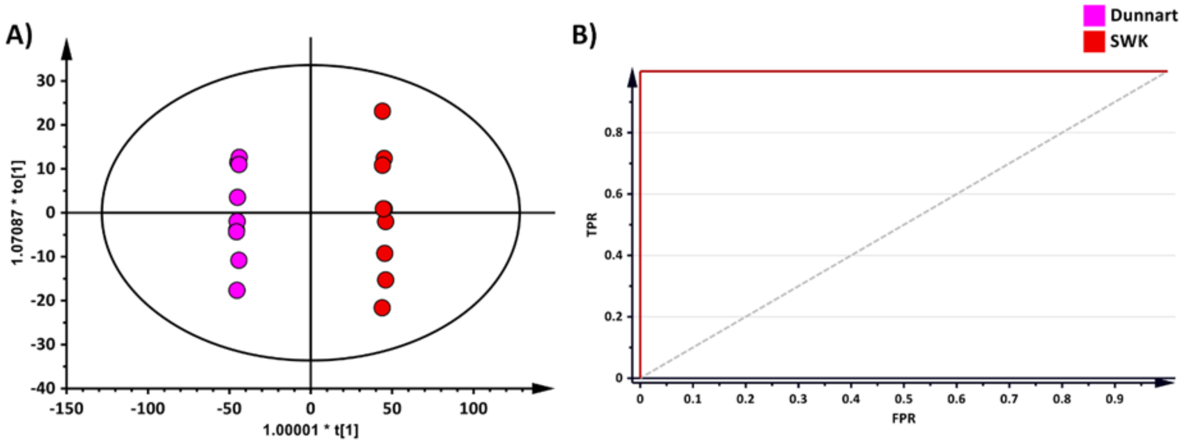

Figure S1. An orthogonal projection to latent structures discriminant analysis (OPLS-DA) model of two representative oat cultivars: Dunnart and SWK001. (**A**) An OPLS-DA scores plot summarizing the relationship among different datasets to visualize group clustering between the Dunnart and SWK001 cultivars based on their leaf-extracted metabolic profiles obtained in ESI (−) MS mode (R2 = 0.998, Q2 = 0.971, CV-ANOVA *p*-value = 5.8941 × 10–14). (**B**) Receiver operator characteristic (ROC) plot for the OPLS-DA model (A) illustrating the performance of the binary classifier, showing perfect discrimination (100% sensitivity and 100% specificity) as the curve passes through the top left corner.

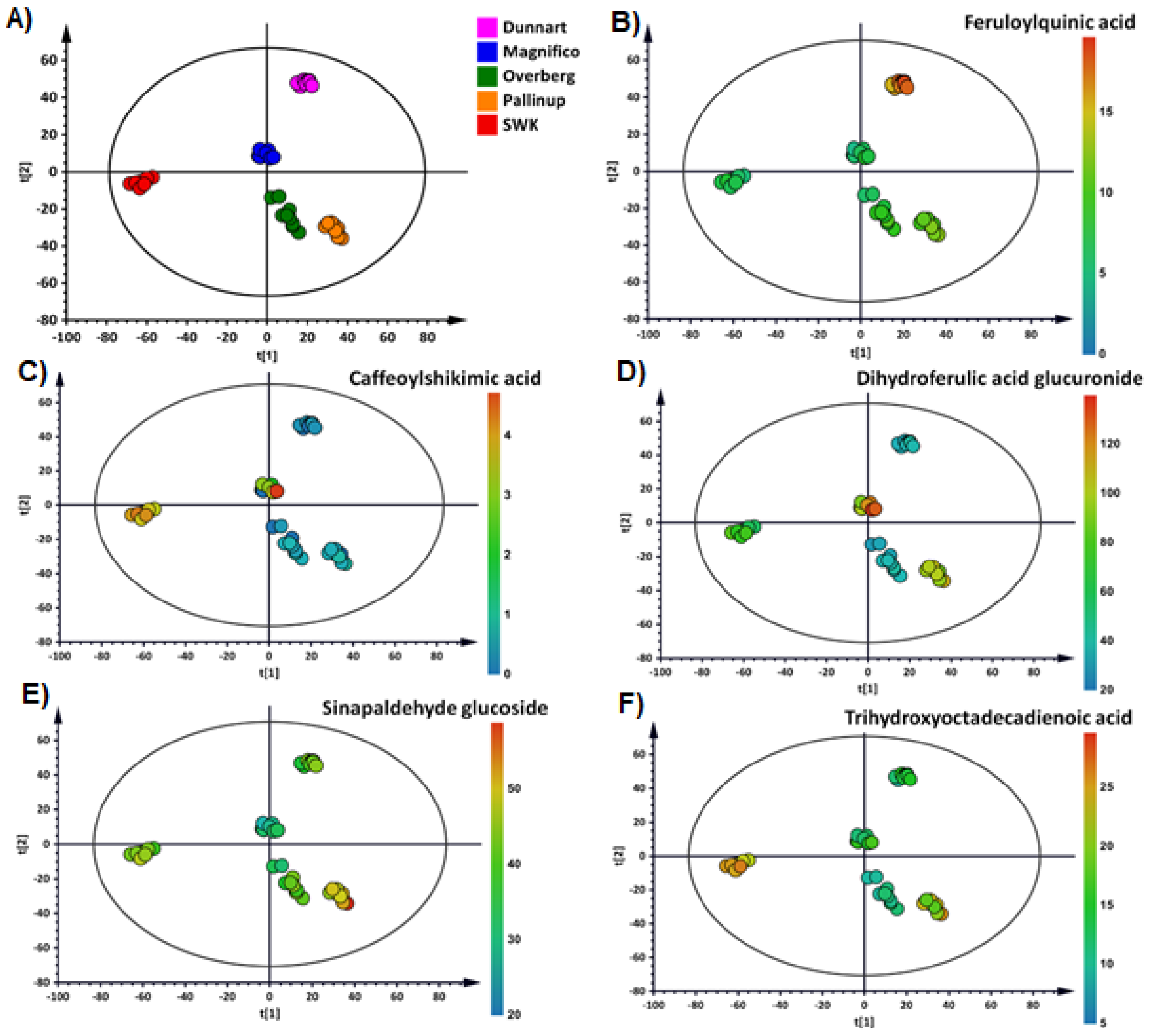

Figure S2. Colour-coded PCA score plots showing the presence and increasing abundance of discriminatory ions in the respective cultivars Overberg, Pallinup, Dunnart, Magnifico & SWK001 (red—high abundance, blue—low abundance). (**A**) PCA scores plot, (**B**) Feruloylquinic acid, (**C**) Caffeoylshikimic acid, (**D**) Dihydroferulic acid glucuronide, (**E**) Sinapaldehyde glucoside and (**F**) Trihydroxyoctadecadienoic acid.
